# Development and multi-center clinical trials of an up-converting phosphor technology-based point-of-care (UPT-POCT) assay for rapid COVID-19 diagnosis and prediction of protective effects

**DOI:** 10.1186/s12866-022-02450-z

**Published:** 2022-02-03

**Authors:** Pingping Zhang, Baisheng Li, Yao Wang, Wei Min, Xiaohui Wang, Yugui Zhou, Zhencui Li, Yong Zhao, Huan Zhang, Min Jiang, Huanying Zheng, Chao Yang, Wei Zhang, Le Zuo, Qi Gao, Zhengrong Yang, Yanzhao Li, Tiejian Feng, Changqing Lin, Qinghua Hu, Tie Song, Ruifu Yang

**Affiliations:** 1 State Key Laboratory of Pathogen and Biosecurity, Beijing Institute of Microbiology and Epidemiology, Beijing, 100071 People’s Republic of China; 2 Beijing Key Laboratory of POCT for Bioemergency and Clinic (No. BZ0329), Beijing, 100071 People’s Republic of China; 3 Guangdong Provincial Center for Disease Control and Prevention, Guangzhou, 511430 People’s Republic of China; 4 Department of Clinical Laboratory, Peking Union Medical College Hospital, Peking Union Medical College, Chinese Academy of Medical Sciences, Beijing, 100032 People’s Republic of China; 5 Shenzhen Center for Disease Control and Prevention, Shenzhen, People’s Republic of China; 6 Guizhou Medical University, Guiyang, People’s Republic of China

**Keywords:** Up-conversion phosphor technology, COVID-19, RBD-specific total antibodies, Clinical trial, Neutralizing activity, Protective effects

## Abstract

**Background:**

Quantitative point-of-care testing assay for detecting antibodies is critical to COVID-19 control. In this study, we established an up-conversion phosphor technology-based point-of-care testing (UPT-POCT), a lateral flow assay, for rapid COVID-19 diagnosis, as well as prediction of seral neutralizing antibody (NAb) activity and protective effects.

**Methods:**

UPT-POCT was developed targeting total antibodies against the receptor-binding domain (RBD) of SARS-CoV-2 spike protein. Using ELISA as a contrast method, we evaluated the quantitation accuracy with NAb and serum samples. Cutoff for serum samples was determined through 70 healthy and 140 COVID-19 patients. We evaluated the cross-reactions with antibodies against other viruses. Then, we performed multi-center clinical trials of UPT-POCT, including 782 patients with 387 clinically confirmed COVID-19 cases. Furthermore, RBD-specific antibody levels were detected using UPT-POCT and microneutralization assay for samples from both patients and vaccinees. Specifically, the antibodies of recovered patients with recurrent positive (RP) reverse transcriptase-polymerase chain reaction test results were discussed.

**Results:**

The ratios of signal intensities between the test and control bands on the lateral flow strip, namely, T/C ratios, was defined as the results of UPT-POCT. T/C ratios had excellent correlations with concentrations of NAb, as well as OD values of ELISA for serum samples. The sensitivity and specificity of UPT-POCT were 89.15% and 99.75% for 782 cases in seven hospitals in China, respectively. We evaluated RBD-specific antibodies for 528 seral samples from 213 recovered and 99 RP COVID-19 patients, along with 35 seral samples from inactivated SARS-CoV-2 vaccinees, and we discovered that the total RBD-specific antibody level indicated by T/C ratios of UPT-POCT was significantly related to the NAb titers in both COVID-19 patients (*r* = 0.9404, n = 527; ρ = 0.6836, n = 528) and the vaccinees (*r* = 0.9063, ρ = 0.7642, n = 35), and it was highly relevant to the protection rate against RP (*r* = 0.9886, n = 312).

**Conclusion:**

This study reveals that the UPT-POCT for quantitative detection of total RBD-specific antibody could be employed as a surrogate method for rapid COVID-19 diagnosis and prediction of protective effects.

**Supplementary Information:**

The online version contains supplementary material available at 10.1186/s12866-022-02450-z.

## Introduction

As of April 9, 2021, over a hundred million cases and over two million deaths were reported during the pandemic of coronavirus disease 2019 (COVID-19), which is caused by a novel coronavirus named severe acute respiratory syndrome coronavirus 2 (SARS-CoV-2) with high infectiousness and mortality rate [1]. Although PCR is becoming a principal method of hospital diagnosis due to its high sensitivity, false RNA-negative results were still detected in a significant number of COVD-19 patients in the practical application in the early stage of COVID-19 [2], and fewer viral loads in the throat swab as normal sample collecting mean than the lower respiratory tract [3]. Therefore, from the seventh edition of Guidelines for Diagnosis and Treatment for Novel Coronavirus Pneumonia issued by the National Health Commission of the people’s Republic of China, the detection of specific antibodies was used as independent evidence to diagnose suspected cases as confirmed cases. Apart from classic laboratory procedures, such as enzyme-linked immunosorbent assay (ELISA) and chemiluminescence assay, point-of-care testing approaches for monitoring and detecting antibodies against SARS-CoV-2 are urgently needed. An up-conversion phosphor technology-based point-of-care testing (UPT-POCT), based on up-conversion phosphor nanoparticles as labels, is an effective method for quantitative biological testing [4–7]. Besides diagnosis, the quantification detection of neutralizing antibodies (NAbs) can also provide a hint for the protective effects of COVID-19, which has been proven in nonhuman primates [8, 9], and it will be more achievable if the complex neutralization test is substituted by POCT methods. NAb that inhibits the binding of the receptor-binding domain (RBD) on the S1 subunit of spike protein to angiotensin-converting enzyme 2 (ACE2) has attracted a lot of attention [10, 11], and NAbs that target the RBD site with significant neutralization potency have been isolated [10, 12]. Although RBD-specific IgG titers are partly related to SARS-CoV-2-specific NAb titers [13], the significance of NAbs that inhibit RBD–ACE2 binding for the overall protective effects cannot be elucidated because other antibodies, such as IgA and IgM [14], may also contribute to the measured NAb titers and viral persistence [15]. Therefore, the total RBD-specific antibody should be the target for POCT methods for diagnosis, prediction of neutralizing activity, and the protective effects of NAbs.

For COVID-19 diagnosis, this study first developed and comprehensively evaluated the UPT-POCT method to quantitatively detect the total antibodies against RBD, using the RBD fragment as the coating protein on its testing strip and S1 protein as the UCP-labeling antigen. We evaluated the diagnostic performance of UPT-POCT in the hospital settings for 782 cases. Furthermore, we analyzed the level of RBD-specific antibodies detected by UPT-POCT and the neutralizing activity of 528 seral samples from 213 recovered and 99 recurrent positive (RP; as defined by polymerase chain reaction [PCR] results) COVID-19 patients, as well as 35 vaccinees, to determine whether UPT-POCT could be an effective surrogate method for quick prediction of the neutralizing activity and protective effects against COVID-19.

## Results

### Development of UPT-LF assay for detecting total antibodies against SARS-CoV-2

**Fig. 1:**
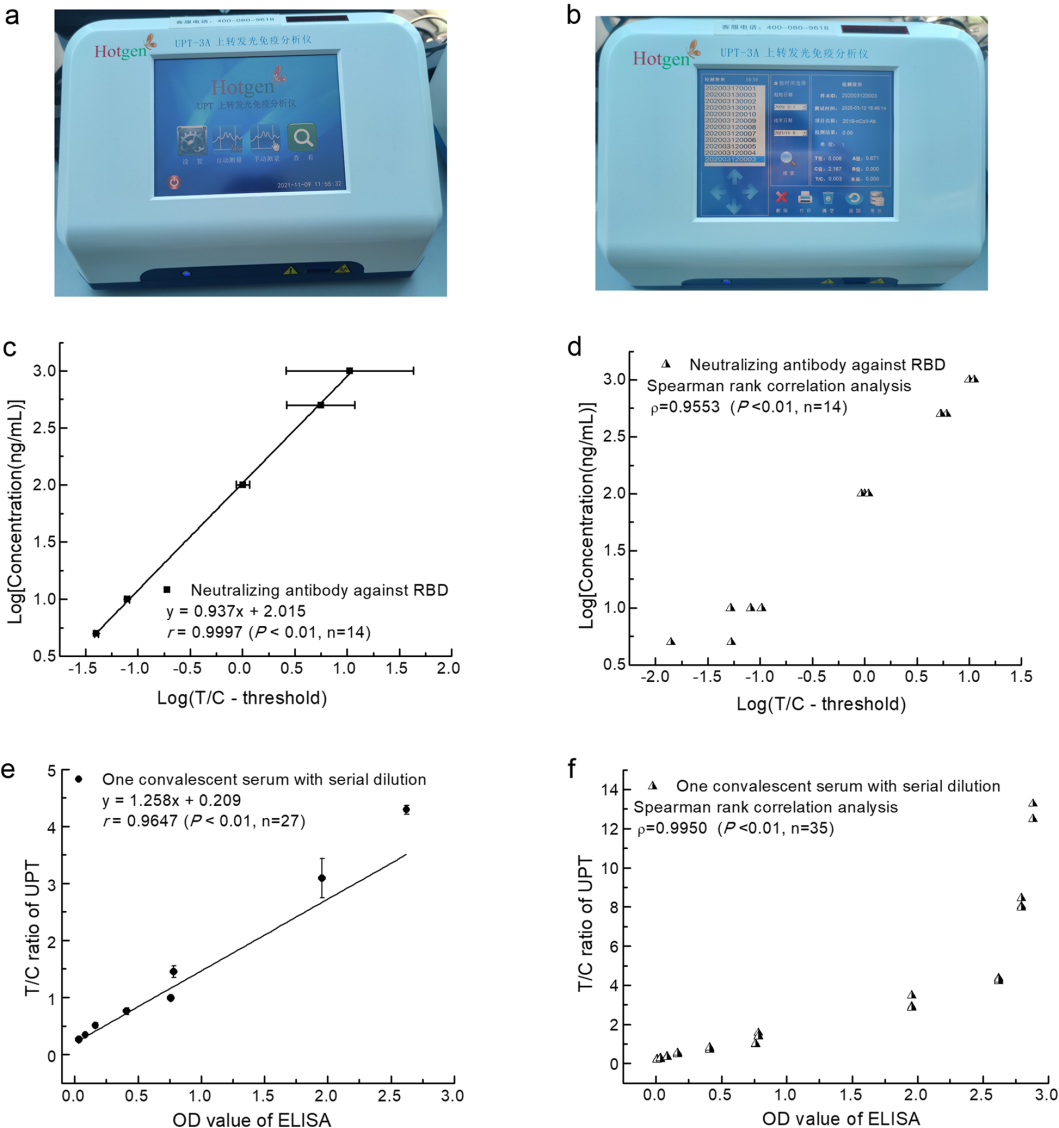
Illustration of UPT biosensor and evaluation of the detection limit of UPT-POCT method. **a** Appearance of UPT biosensor employed in this study. **b** Illustration of result display of UPT biosensor. **c** and **d** are the results for neutralizing antibodies against RBD separated from a convalescent patient from 5 to 1000 ng/mL (*n* = 14). **c** Standard curve of UPT-POCT for quantification. **d** The Spearman correlation analysis between the logarithm of T/C minus methodological threshold and the logarithm of the concentration. **e** and **f** are the results for serially diluted serum samples from another convalescent COVID-19 patient. The linear relationship (**e**) and Spearman correlation (**f**) between ELISA OD value and T/C ratio of UPT

The recombinant proteins, rS-RBD-Fc, and rS1-His were expressed in eukaryotic cells based on the RNA sequence of SARS-CoV-2 strain from Wuhan, China (GenBank accession MN908947). We established a double-antigen sandwich-format UPT strip for detecting total antibodies against SARS-CoV-2, namely, COVID-19-UPT-LF. The test (T) and control (C) lines of nitrocellulose (NC) membrane were coated with 1 mg/ml rS-RBD-Fc and 2 mg/ml goat anti-rS-RBD-Fc polyclonal antibody. Up-conversion phosphor (UCP) particles with amino and aldehyde groups were covalently conjugated with rS1 of 20 μg/ml, forming conjugated UCP-rS1 complexes. For positive samples, the anti-spike antibody in the detected serum is firstly combined with UCP-rS1 and then captured by T line forming [rS-RBD-Fc]- [anti-spike antibody]-[rS1-UCP] when the sample is flowing forward. Regardless of whether the sample is positive or negative, [goat anti-rS-RBD-Fc polyclonal antibody]-[rS1-UCP] will be generated on the C line. For signal acquisition, UCP particles on the T and C lines were excited by the biosensor’s laser diode (Fig. 1a and b), and the ratio of the signal peak value of T and C, namely, T/C, is defined as the result of the detection. T/C ratio is positively proportional to the anti-spike antibody in the positive human serum sample.

### The detection limit of UPT-POCT and its accuracy for quantifying RBD-specific antibodies

We evaluated the detection limit of UPT-POCT by neutralizing antibodies against RBD separated from a convalescent patient (Fig. 1c and d), as well as serially diluted serum samples from another convalescent patient (Fig. 1e and f). First, we analyzed neutralizing antibodies against RBD (Yikang, China) with neutralization potency in the pM range by UPT-POCT. Sample-treating buffers were measured five times and the mean plus three folds of standard deviation was set as a methodological threshold, and the threshold was 0.332. The lowest concentration with at least one of the three tests was defined as the detection limit, and it was 1 ng/ml, while three results were positive for 5 ng/ml. We plotted a standard curve of UPT-POCT for NAb against RBD from 5 to 1000 ng/mL (r = 0.9997, *P* < 0.01, n = 14) (Fig. 1c), Spearman correlation coefficient for the logarithm of T/C minus methodological threshold and the logarithm of the concentration is 0.9553 (*P* < 0.01, n = 14) (Fig. 1d), indicating excellent quantitation precision. Second, data from 25- to 1600-fold diluted seral samples from a convalescent COVID-19 patient indicate that the UPT-POCT T/C ratios are significantly associated with ELISA OD values (y = 1.258x + 0.209, *r* = 0.9647, n = 27) (Fig. 1e). Spearman correlation coefficients is 0.9950 for data ranging from 6.25- to 3200-fold diluted seral samples (*P* < 0.01, n = 35) (Fig. 1f). Besides the similar accuracy for quantification as ELISA, UPT-POCT could also quantitatively analyze the samples diluted 3200-, 12.5-, or 6.25-fold, indicating that this method has a lower detection limit and wider detection range than ELISA (Fig. 1C and Table S1 of Additional file 1).

### Comparison between UPT-POCT and ELISA

**Fig. 2:**
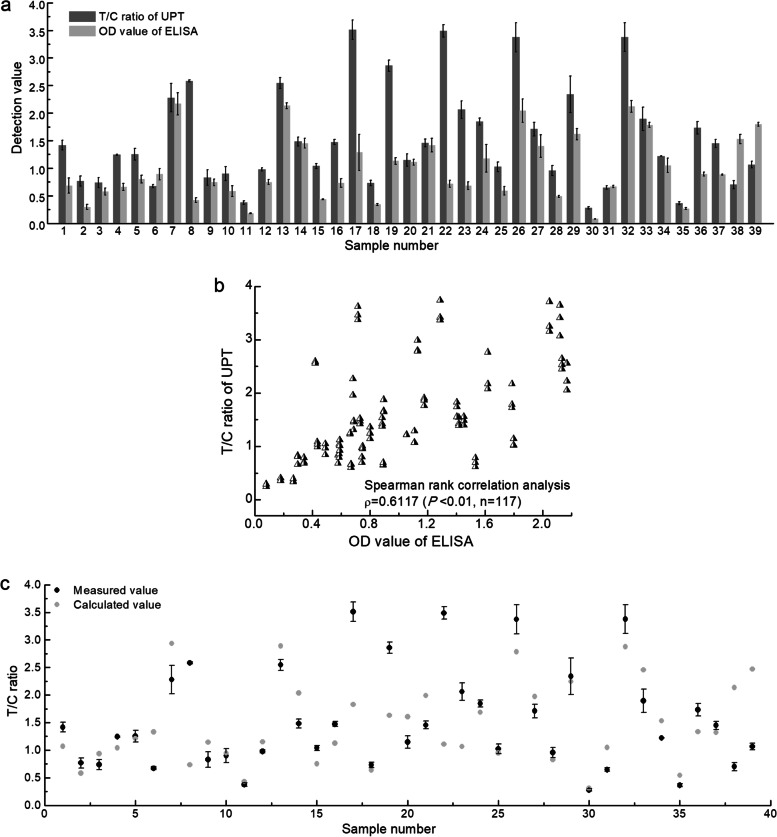
Comparison between UPT-POCT and ELISA. Thirty-nine serum samples were extracted from 27 COVID-19 patients during the acute phase of illness. Twenty-five-fold dilution was for sample No. 2, 400 folds dilution for seven samples (No. 4, 8, 12, 14, 15, 34, and 37), and 100 folds dilution for the remaining 31 samples. Each sample was tested three times using the two methods. **a** Detection results of UPT-POCT and ELISA. **b** Spearman correlation test was used to analyze the relationship between the two methods. **c** Comparison of the measure values of UPT-POCT (Black dots) and the T/C ratio calculated based on ELISA OD value according to the parameters (y = 1.258x + 0.209) of Fig. 1D (Light gray dots)

We estimated the antibody levels in another 39 serum samples from 27 COVID-19 patients during the acute phase (7 – 10 days) of infection using ELISA and UPT-POCT (Fig. 2 and Table S2 of Additional file 1). Spearman rank correlation coefficient between T/C ratio of UPT-POCT and OD value of ELISA is 0.6117 (*P* < 0.01, n = 117) (Fig. 2b). We calculated the T/C ratios by substituting the ELISA OD values for these 39 serum samples into the formula (y = 1.258x + 0.209) of Fig. 1D, the values derived were not significantly different from the measured values, according to a paired *t*-test analysis (*p* > 0.05) (Fig. 2c). This further verifies the accuracy of the UPT-POCT method.

### Cutoff for detecting serum sample and specificity of UPT-POCT

To determine the UPT-POCT cutoff for detecting serum samples, we included 210 samples from 70 healthy people and 140 COVID-19 patients. The cutoff was defined as 0.25, when the maximum Yorden index was 0.971 with sensitivity and specificity of 98.6% and 99.86%, respectively. The area under the ROC curve is 0.989 (95%CI: 0.974 – 1.000), demonstrating the excellent authenticity of diagnosis by UPT-POCT assay.

**Fig. 3:**
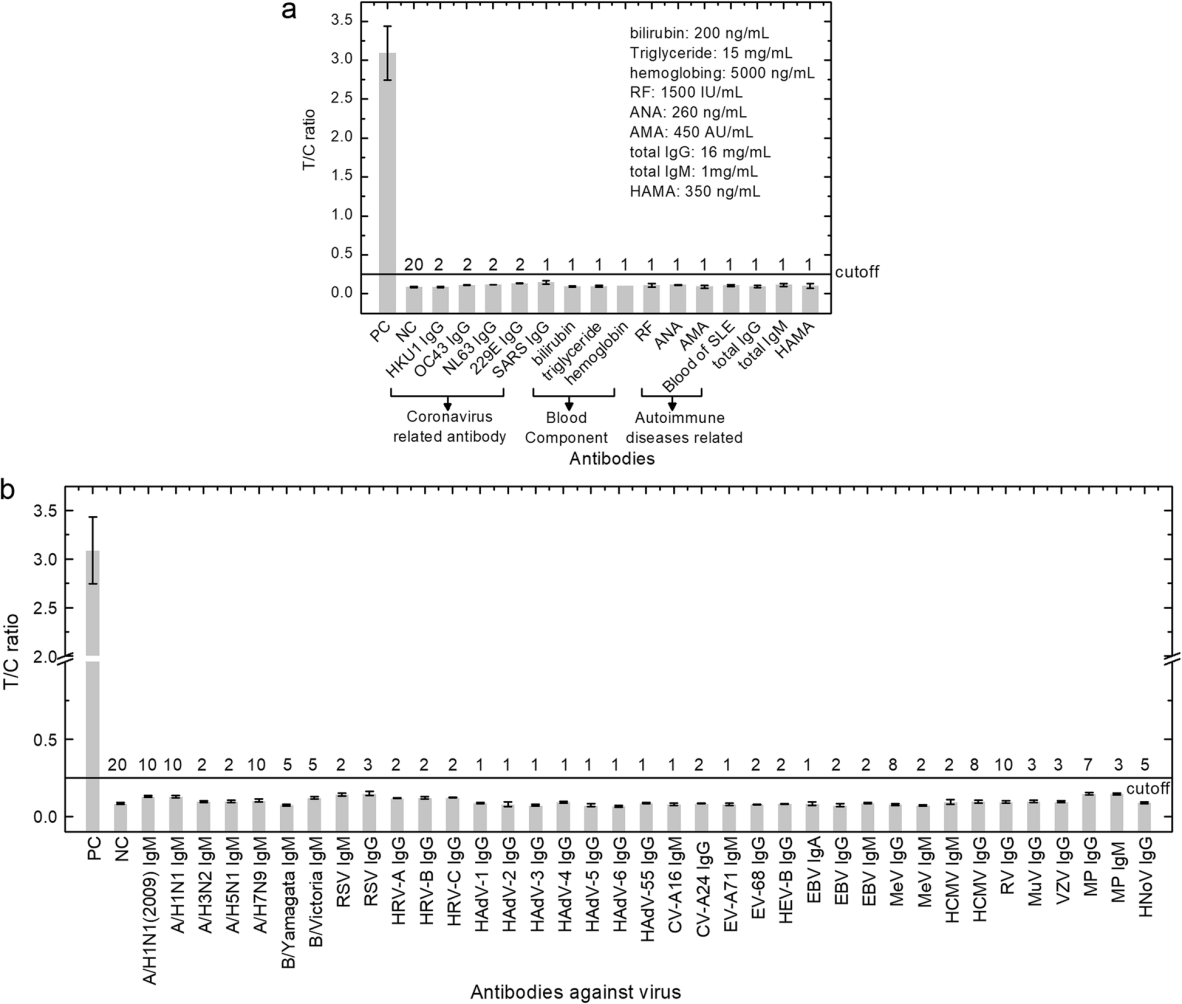
Specificity of UPT-POCT. **a** Specificity of UPT-POCT for antibodies against other coronaviruses, blood components, autoimmune diseases related antibodies, etc. PC, Positive Control, namely, a convalescent serum with 50-fold dilution; NC, Negative control, health people serum; RF: rheumatoid factor; ANA: antinuclear antibody; AMA: anti-mitochondrial antibody; SLE: systemic lupus erythematosus; HAMA: human anti-mouse antibody. **b** Specificity of UPT-POCT for antibodies against other viruses, including A/H1N1(2009), A/H1N1(2009) influenza virus; A/H1N1(or H3N2, H5N1, H7N9), A/H1N1 (or H3N2, H5N1, H7N9) influenza virus; B/Yamagata (or B/Victoria), B/Yamagata (or B/Victoria) influenza virus; RSV, respiratory syncytial virus; HRV-A (or -B, −C), human rhinovirus species A (or B, C); HAdV-1(or − 2, − 3, − 4, − 5, − 6, − 55), human adenovirus type 1(or 2, 3, 4, 5, 6, 55); CV-A16 (or -A24), coxsackievirus 16 (or 24); EV-A71 (or -A68), human enterovirus 71 (or 68); HEV-B: human enterovirus B; EBV, epstein-barr virus; MeV, measles virus; HCMV, human cytomegalovirus; RV, rotavirus; MuV, mumps virus; VZV, varicella-zoster virus; MP, *mycoplasma pneumoniae*; HNoV, Human norovirus. The number above each column is the number of samples. Each sample was tested three times, while the average value was exhibited if the number of samples was more than one

UPT-POCT shows excellent specificity for five antibodies against other coronaviruses except for SARS-CoV-2, three blood components, four autoimmune diseases-related antibodies, or blood samples, total IgG, total IgM, and human anti-mouse antibody (Fig. 3a), and antibodies against other viruses (Fig. 3b).

### Diagnostic performance of UPT-POCT in the hospital settings

From the analysis of 782 serum samples from seven hospitals in China with 387 clinically confirmed COVID-19 patients (Table 1), the sensitivity and specificity of the UPT-POCT is 89.15% (345/387, 95%CI: 85.65–91.87%) and 99.75% (394/395, 95%CI: 98.58–99.96%), respectively. The total consistent rate between UPT-POCT and the clinically confirmed cases is 94.50% (95%CI: 92.68–95.89%), with a Kappa value of 0.89. For the 387 COVID-19 patients, according to the time of symptom onset, 32, 70, and 243 patients were in the early (≤7 days), middle (8–14 days), and late course (≥15 days), while UPT-POCT reveals detection rate of 81.40 and 98.78% in middle and late courses, respectively (Table 2).

**Table 1:** Sensitivity and specificity of UPT-POCT SARS-CoV-2 antibody test

Groups		COVID-19		Total
		the number of positive cases	the number of negative cases	
UPT-POCT	the number of positive cases	345	1	346
	The number of negative cases	42	394	436
	Total	387	395	782

**Table 2:** The detection rates of UPT-POCT test for patients in different courses

Groups	Clinical confirmed cases	UPT-POCT positive case	
		Cases	Detection rate (95% CI)
1–7 days	55	32	58.18% (45.03–70.26%)
8–14 days	86	70	81.40% (71.89–88.21%)
> 14 days	246	243	98.78% (89.15–91.87%)
Total	387	345	89.15% (85.65–91.87%)

### Relationship between the total RBD-specific antibody level and NAb titers

**Fig. 4:**
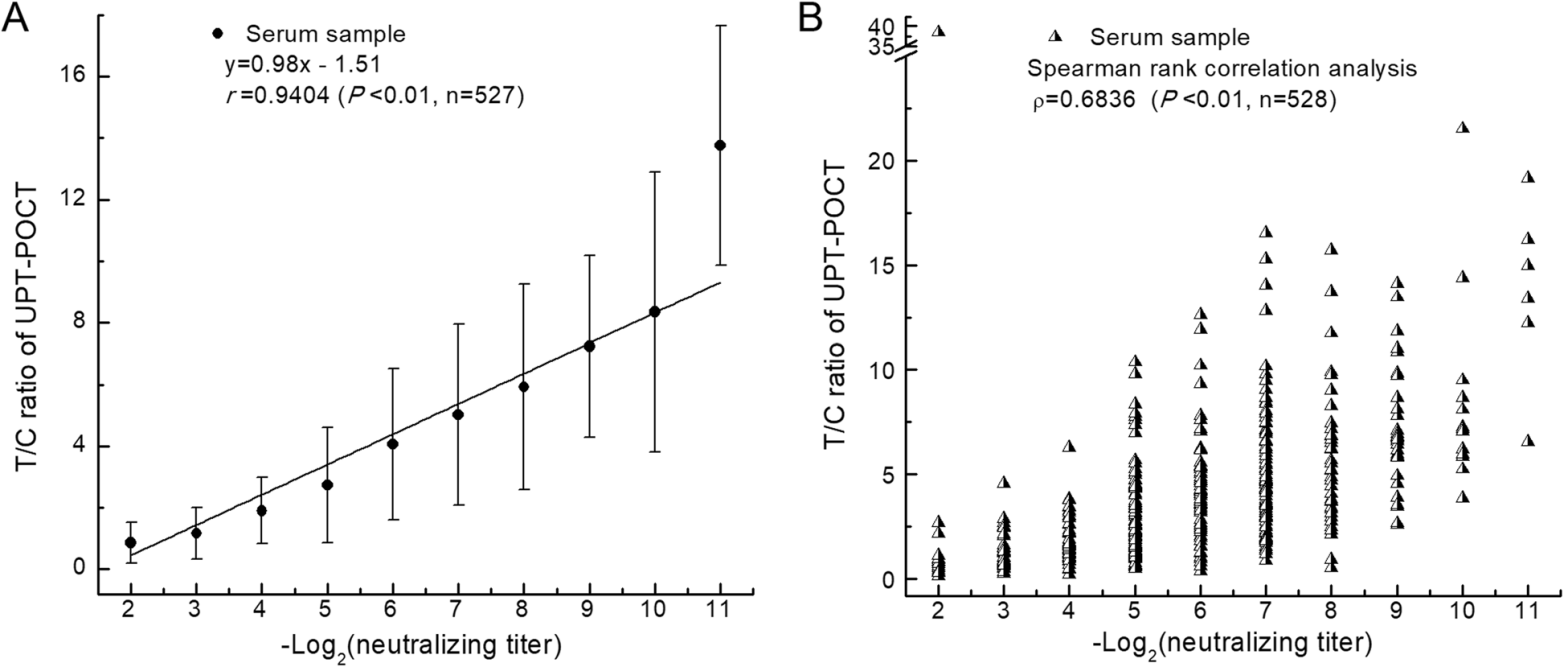
Correlation between the T/C ratios of UPT-POCT and NAb titers in 528 serum samples. The corresponding relation between -Log_2_(neutralizing titer) and neutralizing titer: 2 (1:4), 3 (1:8), 4 (1:16), 5 (1:32), 6 (1:64), 7 (1:128), 8 (1:256), 9 (1:512), 10 (1:1024), 11 (> 1:1024). **a** Linear regression analysis (n = 527). The error bar for neutralizing titer ranging from 1:4 to > 1:1024 is derived from the detection results for 16, 46, 54, 140, 75, 103, 40, 33, 14, and 6 samples, respectively. **b** Spearman correlation analysis (n = 528). Compared with linear regression analysis, we included a sample with a neutralizing titer of 1:4 and a T/C ratio of 38.54

The above-mentioned results indicate that the UPT-POCT assay can be used to accurately quantify the total RBD-specific antibodies in serum. Therefore, for the following text, the UPT-POCT T/C ratio is taken to indicate the total RBD-specific antibody level. For 812 serum samples from 590 participants recruited from Guangdong Provincial Center for Disease Control and Prevention (Guangdong CDC), the number of negative and positive serum samples were 284 and 528, respectively. The Spearman and linear correlation between the T/C ratios and the NAb titers were calculated using the positive serum samples (Fig. 4 and Table S3 of Additional file 1). As shown in Fig. 4, the T/C ratios, which represent the total RBD-specific antibody levels, were significantly related to the NAb titers ranging from 1:4 to > 1:1024, with linear and Spearman rank correlation coefficient of *r* = 0.9404 (*p* < 0.01, n = 527) and ρ = 0.6836 (*P* < 0.01, n = 528). Thus, the total RBD-specific antibody level, indicated by the UPT-POCT T/C ratio, could be used to evaluate the potential protective effects against COVID-19. Most (60.2%, 318/528) of the serum samples had NAb titers between 1:32 and 1:128, corresponding to T/C ratios of 2.735 ± 1.860 to 5.019 ± 2.941.

### UPT-POCT for predicting the protective effects in COVID-19 patients

**Fig. 5:**
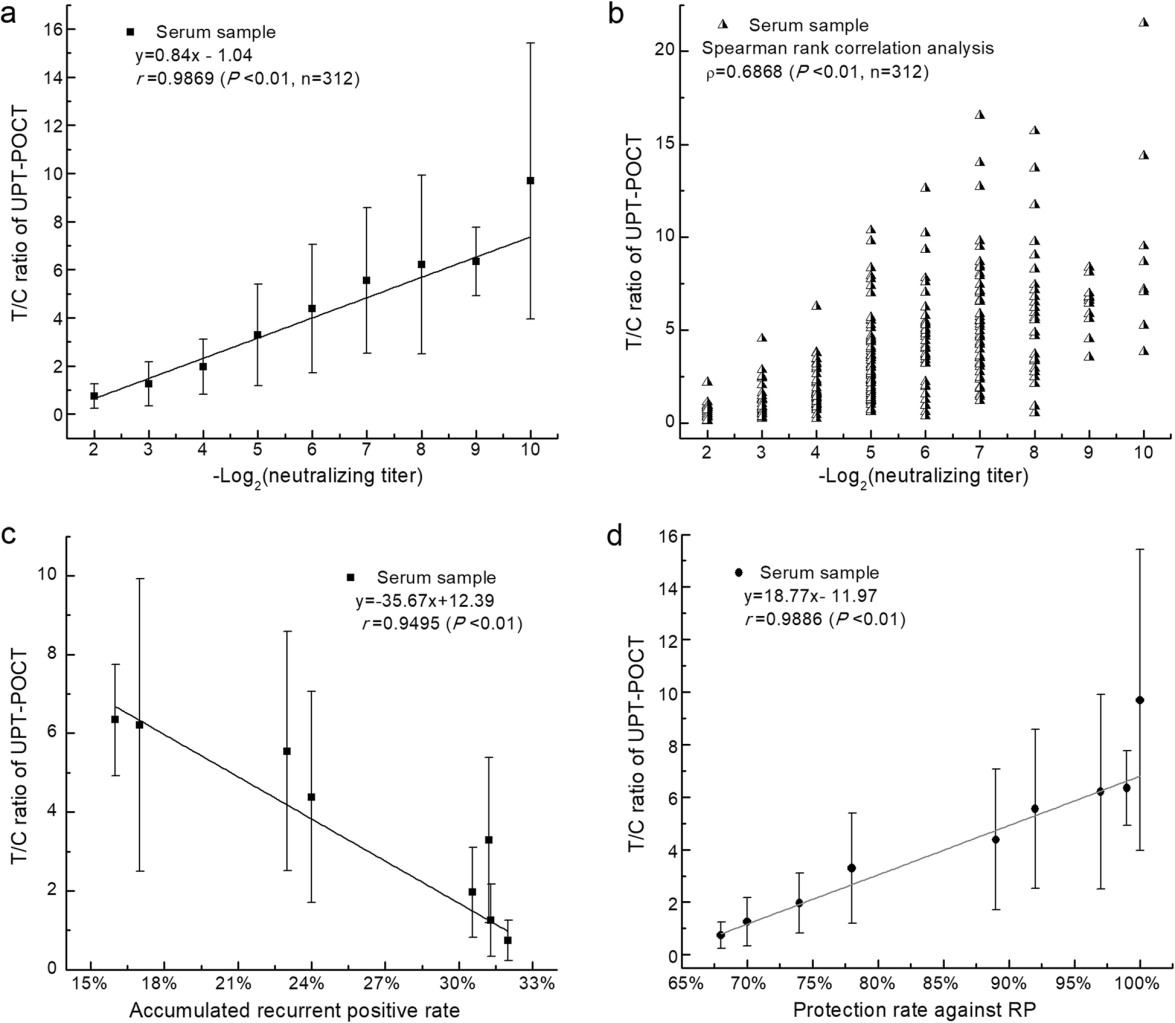
Correlations between T/C ratios, Nab titers, and protection rates for 312 COVID-19 patients. T/C ratio is taken to indicate the total RBD-specific antibody level. The corresponding relation between -Log_2_(neutralizing titer) and neutralizing titer: 2 (1:4), 3 (1:8), 4 (1:16), 5 (1:32), 6 (1:64), 7 (1:128), 8 (1:256), 9 (1:512), 10 (1:1024). All patients were discharged from hospitals of Guangdong according to the criteria and quarantined for 14 days by Guangdong CDC. If the SARS-CoV-2 RNA test by PCR is positive in this period, the case is defined as recurrent-positive, namely, without protection. Linear (**a**) and Spearman (**b**) correlation between T/C ratios of UPT-POCT and NAb titers in 312 serum samples from COVID-19. Linear regression between the T/C ratios and accumulated recurrent positive rate (**c**) or protection rates against RP (**d**). The error bar for neutralizing titers ranging from 1:4 to 1:512 or 1:1024 is derived from the detection results for 15, 32, 44, 77, 41, 57, 27, 11, and 8 samples, respectively

We investigated the relationships between neutralizing titers, RP rates, accumulated RP (ARP) rates, and protection rates against RP for 312 COVID-19 patients among the above-mentioned 590 participants recruited from Guangdong CDC (Fig. 5, Table 3, and Tables S4 – S5). For this study, RP rate was defined as the ratio of RP cases to the sum of RP and NRP patients at each titer [i.e., RP rate = RP/(RP + NRP)], whereas the ARP rate at a specific NAb titer was defined as the ratio of ARP patients to the sum of ARP and accumulated NRP patients at titers above the designated titer [i.e., ARP rate = ARP/(ARP + accumulated NRP)]. We collected serial serum samples from patients, and some had more than one neutralization result; for these cases, the lowest NAb titer and its corresponding T/C ratio were selected for further analysis. Consequently, 312 cases, including 213 recovered (98 male and 115 female) and 99 RP (58 male and 41 female) COVID-19 patients were included, with a median age of 40 years (range: 10 months to 86 years old). Out of 99 RP patients, 17, 75, 4, and 3 are asymptomatic, mild, severe, and critical cases, respectively. For these 312 cases, linear and Spearman rank correlation coefficient of *r* = 0.9869 (*p* < 0.01, n = 312) and ρ = 0.6868 (*P* < 0.01, n = 312) were discovered between T/C ratios and the logarithm of neutralizing titers (Fig. 5a and b).

**Table 3:** The correlation between RP rates, accumulated RP rates, protection rates, and T/C ratios

RP rate for each titer					Accumulated RP (ARP) rate					Protection rate		T/C ratio	
Neutralizing Titer	NRP cases	RP cases	Sum	RP rate ^**a**^	Neutralizing Titer	Accumulated NRP cases	Accumulated RP cases	Sum	ARP rate ^**b**^	Total –Accumulated RP	Protection rate^**c**^	MEAN	SD
**1:4**	9	6	15	40%	≥1:4	213	99	312	32%	213	68%	0.738	0.509
**1:8**	20	12	32	38%	≥1:8	204	93	297	31%	219	70%	1.246	0.914
**1:16**	32	12	44	27%	≥1:16	184	81	265	31%	231	74%	1.960	1.143
**1:32**	42	35	77	45%	≥1:32	152	69	221	31%	243	78%	3.287	2.102
**1:64**	31	10	41	24%	≥1:64	110	34	144	24%	278	89%	4.380	2.677
**1:128**	41	16	57	28%	≥1:128	79	24	103	23%	288	92%	5.546	3.033
**1:256**	22	5	27	19%	≥1:256	38	8	46	17%	304	97%	6.204	3.71
**1:512**	8	3	11	27%	≥1:512	16	3	19	16%	309	99%	6.339	1.414
**1:1024**	8	0	8	0	≥1:1024	8	0	8	0%	312	100%	9.689	5.73
**Total**	213	99	312										

^a, b^ RP and ARP rates is calculated as the ratio of the number of RP cases (or accumulated cases) to the sum of RP and NRP patients (or accumulated cases) at each specific titer

^c^ Protection rate against RP = (312- accumulated RP cases)/312

As shown in Table 3 and Table S4 of Additional file 1, at T/C ratios of ≥ 4.380 ± 2.677 (corresponding to NAb titers of ≥1:64), the RP rates at each NAb titer steadily decreased and remained below 30%, with half of all cases (46%, 144/312) meeting those criteria. The correlation between total RBD-specific antibody levels (T/C ratios) and ARP rates is illustrated in Fig. 5c, as well as Table 3 and Table S5 of Additional file 1. Overall, the total RBD-specific antibody level (T/C ratio) was strongly inversely related to ARP rate (*r* = 0.9495, *p* < 0.01, n = 312). As with the RP rates, at T/C ratios of ≥ 4.380 ± 2.677 (corresponding to NAb titers of ≥ 1:64), ARP rates showed a constant decline from 24% (34/144) to 0%, covering almost half (46%, 144/312) of all cases. For the remaining patients (54%, 168/312), with NAb titers below 1:64, the ARP rate was approximately 30%. Additionally, RP rates at T/C ratios in the range of 4.380 ± 2.677 to 9.689 ± 5.730 (corresponding to NAb titers in the range of 1:64 to 1:1024) (24%, 34/144; namely RP/Total) were significantly lower than those at T/C ratios in the range of 0.738 ± 0.509 to 3.287 ± 2.102 (corresponding to NAb titers in the range of 1:4 to 1:32) (39%, 65/168; namely RP/Total). These results are consistent with the idea that high total RBD-specific antibody levels, like high NAb titers, can protect recovered COVID-19 patients against becoming RP.

The relationship between total RBD-specific antibody levels (T/C ratio) and protection rates against was investigated (Fig. 5d, Table 3, and Table S6 of Additional file 1). The protection rate against RP at each NAb titer was defined here as the ratio of the total number of cases minus the number of APR patients to the total number of cases [(total cases − ARP patients)/total cases]. As shown in Fig. 5d and Table 3, protection rates reached 89% and 78% for T/C ratios of ≥ 4.380 ± 2.677 (corresponding to NAb titers of ≥1:64) and 3.287 ± 2.102 (corresponding to NAb titers of ≥ 1:32), covering approximately half (46%, 144/312) and three quarters (71%, 221/312) of total cases, respectively. Notably, protection rates still exceeded 68% for T/C ratios of ≥ 0.738 ± 0.509 (corresponding to NAb titers of ≥ 1:4), demonstrating that, as long as they exist, the antibodies can provide significant protection for recovered COVID-19 patients against becoming RP. A linear regression analysis on the total RBD-specific antibody levels (T/C ratios) and protection rates against RP was performed, yielding a correlation coefficient of *r* = 0.9886 for T/C ratios in the range of 0.738 ± 0.509 to 9.689 ± 5.730 (corresponding to NAb titers in the range of 1:4 to 1:1024) and protection rates against RP ranging from 68% to 100%. This reveals that the total RBD-specific antibody level, indicated by UPT-POCT T/C ratio, has value for predicting protection rates against RP in COVID-19 patients.

### UPT-POCT for predicting the protective effects in vaccinated individuals

**Fig. 6:**
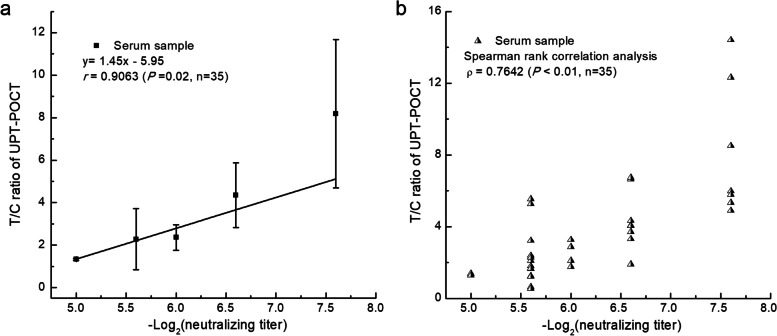
Correlation between the total RBD-specific antibody levels and NAb titers in vaccinated individuals. (**a**) Results of linear correlation analysis. (**b**) Results of Spearman rank correlation analysis. Here, the left y-axis presents the T/C ratios reported by UPT-POCT, which are taken to indicate the total RBD-specific antibody level. The corresponding relation between -Log_2_(neutralizing titer) and neutralizing titer: 5 (1:32), 5.6 (1:48), 6 (1:64), 6.6 (1:96), 7.6 (1:192)

Thirty-five serum samples with NAb titers of 1:32 to 1:192 were selected for analyzing the correlation between the total RBD-specific antibody level and neutralizing activity after COVID-19-vaccination (Fig. 6 and Table S7 of Additional file 1). As shown in Fig. 6, the total RBD-specific antibody levels (T/C ratios) were significantly associated with the NAb titers by linear correlation (*r* = 0.9063, *P* = 0.02, n = 35) and Spearman rank correlation (ρ = 0.7642, *P* < 0.01, n = 35) analysis, revealing that the total RBD-specific antibody level, indicated by UPT-POCT T/C ratio, has value for predicting the levels of protection rates against RP in vaccinated individuals.

## Discussion

Here, a quantitative point-of-care testing, UPT-POCT, was first developed for diagnosis and prediction of protective effects. The proliferation of SARS-CoV-2 can occur at any time of year and the current pandemic has been predicted to last for several years [16], while disease-induced immunity against SARS-CoV-2 is believed to persist for months [17], and anti-RBD antibody titers are related to viral persistence [15]. Although RBD-specific antibodies are considered to comprise most protective NAbs, the rapid method for quantitative evaluation of the level of total RBD-specific antibodies and neutralizing activity has not been well developed. By mixing serum with sample-treating buffer and applying the sample to the strip for 15 min reaction, UPT-POCT can provide detection results, which are faster, safer, and easier to operate than ELISA. The detection limit for neutralizing antibody against RBD is 1 ng/mL, and the sensitivity and specificity of UPT-POCT is 89.15% and 99.75% for 782 cases from seven hospitals. For 528 serum samples from 213 recovered and 99 RP COVID-19 patients, the levels of total antibodies against RBD indicated by T/C ratios of UPT-POCT were significantly associated with the neutralizing activity and protection rates against RP. The high correlation between total RBD-specific antibody levels and NAb titers was further verified in vaccinated individuals. This could help assess the effectiveness of COVID-19 vaccination, the utility of convalescent plasma therapy, and the feasibility of herd immunity and immunity passports, and other preventive and control measures against COVID-19.

This study discovered some important points regarding antibody-based protection against COVID-19 for discharged patients from hospitals. First, the most common level of neutralizing activity found in serum samples of patients was NAb titers in the range of 1:32 to 1:128 (60.2%), which can lead to the risk of becoming RP. Second, a NAb titer of 1:64 was identified as the critical threshold for reducing the risk of becoming RP; therefore, this value is recommended as an indicator for predicting the protective effects of NAb on SARS-CoV-2.

What’s important, the previous study shows the correlation coefficient between RBD-specific IgG or IgA titers and the microneutralization titer can only reach 0.6 or 0.49, respectively [15], which are lower than that of our study. The reasons may be as follows: (1) Total antibodies, including IgG, IgA, etc., were detected by double-antigen sandwich mode in our study, rather than IgG or IgA by indirect method; The results also prove the high diversity of NAbs against RBD. (2) UPT-POCT can give continuous value for detection of samples, which is superior to semi-quantitative results of ELISA in other studies; (3) The number of samples used in this study, 528 sera, is far more than that in other studies.

Our study has limitations. Firstly, our data analysis did not account for the reason an individual became RP. Secondly, although a strong correlation existed between the total RBD-specific antibody level and protection rates against RP in this study, the particular contributions of cellular immune could not be ignored for protective effects but were not included in the analysis. Thirdly, some samples were from patients discharged from hospitals, but the time for hospitalization could not be determined precisely.

## Conclusion

UPT-POCT method for detecting total antibody against SARS-CoV-2 was well established with a sensitivity of 89.15% and specificity of 99.75% (n = 782). The quantitative detection results of UPT-POCT for total antibodies against RBD were significantly correlated with the NAb titers in patients (*r* = 0.9404, n = 527; ρ = 0.6836, n = 528) and vaccinated participants (*r* = 0.9063, ρ = 0.7642, n = 35), as well as with the protection rate against becoming RP (*r* = 0.9886, n = 312). It can be used as a surrogate method for rapid diagnosis and prediction of protective effects.

## Methods

### Participants and specimens

All patients were diagnosed, treated, and discharged according to “Guidelines for Diagnosis and Treatment for Novel Coronavirus Pneumonia (Seventh Edition)” issued by the National Health Commission of the People’s Republic of China. Following the Guidelines, we performed comprehensive analysis for all individuals to determine the clinically confirmed COVID-19 cases in this study, incorporating epidemiological investigations and clinical manifestations, and nucleic acid detection, and so on. Employing China’s Online Reporting System for Infectious Disease Epidemics and Public Health Emergencies, every case of COVID-19 was registered with Centers for Disease Control at each level (municipal, provincial, and national department), and epidemiological investigation revealed that no individual in this study had ever had a previous infection of COVID-19. Leftover samples after all other clinical testing were used for the research in this study. Hospitalized patients were recruited from seven designated hospitals for treating COVID-19 patients in China. Patients in the quarantine period after being discharged from the hospital were recruited from Guangdong CDC. Health persons immunized by the COVID-19 vaccine were recruited by Sinovac Biotech Co., Ltd. (Beijing, China). All participants or legally authorized representatives of minor participants provided written informed consent. To protect patient identity, personal identifiers were removed and replaced with uniquely identifiable bar codes.

The participants and specimens included in this study were divided into six groups (Table 4) for various purposes: (1) Evaluation of limit of detection of UPT-POCT for quantifying the total RBD-specific antibodies; (2) Comparison between UPT-POCT and ELISA method; (3) Definition of a cutoff of UPT-POCT for detecting serum sample; (4) Diagnostic performance of UPT-POCT in hospital settings; (5) analysis of the correlation between the levels of total RBD-specific antibodies and neutralizing activity in 528 serum samples from 312 COVID-19 patients, while the lowest NAb titer for each COVID-19 patient was chosen and used for an analysis of the protective effects; and (6) analysis of the correlation between the levels of total RBD-specific antibodies and neutralizing activity in serum samples from vaccinated individuals with NAb titers higher than 1:32. For the first to the fourth group, serum samples were from patients in seven designated hospitals. For the sixth group, serum samples were provided by health persons vaccinated against COVID-19 recruited by Sinovac Biotech Co., LTD. For the fifth group, 812 COVID-19 patients in Guangdong province were treated at hospitals and reported to Guangdong CDC from February 5 to April 14, 2020. Before discharge, the patients were without respiratory symptoms, maintained normal temperature for above three days, had reports of two SARS-CoV-2 RNA-negative results, with at least one-day intervals, while their pulmonary lesions were substantially absorbed for observation by chest computed tomography (CT). After discharge, the patients were all quarantined for 14 days at centralized facilities according to the organization of Guangdong CDC. If positive results of polymerase chain reaction (PCR) for detecting SARS-CoV-2 RNA were found during this period, they were defined as recurrent-positive (RP) and for further continually monitoring. Additionally, the characteristics of some of these patients were disclosed in the previous study [18].

**Table 4:** The participants and specimens included in this study

Group	Aim	Participants and Specimens			Sample size for each neutralizing titer
		Total samples	Positive	Negative	
1	Detection limit	2	one serum sample and one neutralizing antibody from two convalescent patients, respectively;	0	–
2	Comparison between UPT-POCT and ELISA	39	39 samples, from 27 patients at acute phase (7–10 days)	0	–
3	Definition of cutoff of UPT-POCT for detecting serum sample	210	140 samples, from 140 COVID-19 patients at different courses	70 samples, from 70 healthy people	–
4	Diagnostic performance of UPT-POCT in the hospital settings	782	387 samples, from 387 COVID-19 patients’ samples at different courses	395 samples, from 395 healthy people	–
5	The relationship between RBD-specific total antibodies and neutralizing activity	812	528, from recovered or recurrent patients*	284	≥ 14
	UPT-POCT for predicting the protective effects in COVID-19 patients	312	312 serum samples from 213 recovered and 99 PCR re-positive COVID-19 patients*	0	≥ 8
6	UPT-POCT for predicting the protective effects in vaccinated individuals	35	35 vaccinated individuals with NAb titers equal or higher than1:32	0	≥ 5 (except the titer 1:32)

Note: * Some patients were participated in sample collection for more than one times. However, only one sample for each patient was chosen for use in further analysis of protective effect, namely the sample with the lowest NAb titer

### Regents

Nitrocellulose membranes and glass fiber were obtained from Millipore Sigma (Saint Louis, USA), Absorbent paper and sticky based on the strip were from Goldbio Technology Co. Ltd. (Shanghai, China). UCP particles with excitation and emission peaks of 980 nm and 541.5 nm were obtained from Shanghai Kerune phosphor Technology (Shanghai, China) and modified with amino and aldehyde groups in our laboratory. The portable UPT biosensor manufactured by our laboratory was equipped with a laser diode and photomultiplier tube, which is similar to our previous biosensor [19], which can emit 980 nm light, eradiate visible light signals, and scan the strip to obtain the detection result. Our laboratory prepared the eukaryotic recombinant S1 containing His-tag (rS1-His) and S-RBD protein containing the mouse Fc fragment (rS-RBD-mFc), as well as goat polyclonal antibody, for both UPT-POCT and ELISA, as previously described [20]. Neutralizing antibody against RBD (YK003Ab030) with half-maximal inhibitory concentration (IC_50_) of 0.57 ng/mL [multiplicity of infection (MOI) = 0.05], produced from a lymphocyte cell isolated from a convalescent COVID-19 patient by FACS single-cell sorting, were obtained from Yikang Biopharmaceuticals Co., Ltd. (Suzhou, China).

### Establishment of UPT-POCT assay

The rS-RBD-mFc and goat anti-rS-RBD polyclonal antibodies were dispensed on nitrocellulose membranes as test line (T) and control line (C) at a speed of 1 μL/cm. UCP particles of 1 mg/ml were conjugated with rS1-His proteins at 37 °C for 1 h and poured onto glass fiber after being blocked by bovine serum albumin (BSA) for preparing the conjugation pad. Finally, the nitrocellulose membrane, conjugation pad, sample pad (an empty glass fiber), and absorbent paper adhered on a sticky base for clipping 4 mm pieces and fabrication of strips. For the UPT-POCT assay, a 100-μl mixture composed of 10-μl serum and 90-μl sample-treating buffer was applied to the sample window on the strip. After 15 min of lateral flow, the result was read using a specific biosensor for test and control bands, with results reported as the ratio of the test (T) to control (C) signals (T/C ratio) [19]. For sensitivity optimization, the concentrations of proteins and antibodies in the strip and ingredients of sample treating buffer were modulated to increase the difference in T/C ratio between negative and positive samples.

### ELISA

The enzyme-linked immunosorbent assay (ELISA) based on the double-antigen sandwich model was developed by our laboratory to detect serum samples as a comparison method to UPT-POCT. The ELISA kit included a 96-well plate coated with rS1 protein and horse-radish peroxidase (HRP) labeled rS-RBD-Fc protein. The 50-μl serum sample was added to the pore, and antibodies against SARS-CoV-2 in the positive sample can be captured. And then 50 μl of HRP-labeled rS-RBD-Fc solution was immediately added for incubation at 37 °C for 60 min to form the complex of rS1-antibody- rS-RBD-Fc. Subsequently, the plate was washed by 0.05 mol/L phosphate-buffered saline with 0.5‰ Tween. After 100-μl TMB solution was added for coloration, the reaction was terminated by 2 mol/L H_2_SO_4_. Next, we measured the OD value of each pore using a microplate reader (Bio-rad, USA).

### Evaluation of the detection limit of the UPT-POCT method and its quantitative ability

The detection limit and quantitative ability of the UPT-POCT method was assessed using a highly purified neutralizing antibody (NAb YK003Ab030) and a convalescent-phase serum with a series of concentrations. NAb YK003Ab030 was diluted by sample-treating buffer into 1, 5, 10, 100, 500, 1000 ng/mL for UPT-POCT detection, and each was added to three strips (except two strips for 1000 ng/mL). Five strips were added with sample-treating buffer as a negative control. After 15 min, the T/C ratios of the strips were read by the UPT biosensor. The logarithm of T/C ratios minus threshold and logarithm of concentrations of NAb were used for plotting the standard curve, and we analyzed their Spearman correlation coefficient.

To evaluate the detection limit of the UPT-POCT method with ELISA as a comparison, a serum sample from a convalescent COVID-19 patient was diluted 6.25-, 12.5-, 25-, 50-, 100-, 133-, 200-, 400-, 800-, 1600-, and 3200-fold with normal saline and then subjected to simultaneous testing by the UPT-POCT and ELISA method. The sample for each dilution was tested three times, except 800-fold tested six times and 6.25-fold tested two times. Furthermore, we analyzed the correlation between ELISA OD value and the T/C ratio to determine the accuracy of UPT-POCT for quantifying antibodies.

### Comparison between ELISA and UPT-POCT

Another 39 serum samples were assessed using both ELISA and UPT-POCT to further validate the accuracy of UPT-POCT. Firstly, all serum samples were diluted 100-fold for detection. And then higher or lower dilution folds were applied for the serum samples out of detection range with 100-fold dilution. Ultimately, 31 samples were diluted 100-fold, one sample (No. 2) was diluted 25-fold, and seven samples (Nos. 4, 8, 12, 14, 15, 34, and 37) diluted 400-fold (Table S2 of Additional file 1) for detection. Each sample was tested three times.

### Cutoff for detection of serum samples and specificity of UPT-POCT

Besides the methodological threshold determined by the sample-treating buffer, the practical threshold was set to discriminate the positive and negative serum samples, and it was defined as the cutoff. Two hundred and ten samples from 70 healthy people and 140 COVID-19 patients were detected using UPT-POCT assay. The receiver operating characteristic curve (ROC) and maximum Yorden Index were used to determine cutoff employing the statistics software of SPSS.

The specificity of UPT-POCT was evaluated by many antibodies and the blood components, with a convalescent serum with 50-fold dilution and sera from 20 healthy people as the positive and negative controls, respectively. Each sample was applied three times. If more than one sample was collected for a substance, the average values of T/C ratios for these samples were further calculated for statistics.

### Diagnostic performance in the hospital settings

We conducted multi-center clinical trials to evaluate the performance of UPT-POCT assay at seven hospitals in China, including Beijing Center for Disease Control and Prevention, Peking Union Medical College Hospital, the Third Hospital of Ezhou, Fifth Medical Center of PLA General Hospital, Sixth Hospital of Shenyang City, Fifth Hospital of Shijiazhuang City, and Wuhan Huoshenshan Hospital. UPT-POCT was performed on 782 serum samples, 387 of which were from clinically confirmed COVID-19 patients in different courses, and 395 from non-COVID-19 patients, according to The Diagnosis and Treatment Protocol for the COVID-19 (seventh edition) in China. For UPT-POCT, the sample with a T/C ratio higher than cutoff was judged as positive, otherwise, it was judged as negative. Among the clinically confirmed patients, the ratio of the UPT-positive cases to all confirmed cases was defined as sensitivity. Among the non-COVID-19 patients, the ratio of UPT-negative cases to all non-COVID-19 cases was defined as specificity.

### Microneutralization assays

We performed the microneutralization assay according to the references [21, 22]. After being rinsed with 0.25% trypsin, a Vero-E6 cell suspension of 1 – 2 × 10^4^ cells/100 μl was added to a 96-well plate and cultured for 2 – 3 days to form monolayers. Serum samples were inactivated at 56 °C for 30 min and then diluted two-fold in cell culture maintenance medium (MM) within the wells of the microtiter plate, with each well containing a final volume of 120-μl diluted serum. A solution of SARS-CoV-2 viral strain 20SF014/Vero-E6/3 (GISAID accession number EPI_ISL_403934) with a titer of 10^4.67^ 50% tissue culture infectious dose (TCID_50_)/50 μl was diluted to 100 TCID_50_/50 μL, and then 120-μL diluted viral sample was added to the wells containing serum. After the contents were gently blended, the plates were incubated at 37 °C with 5% CO_2_ for 2 h. Subsequently, 100 μL of each diluted serum and virus sample mixture were added in duplicate to wells containing the Vero-E6 cell monolayers. Four wells containing only cells were used as negative controls. After adding 100-μl cell culture MM, the microplates were covered and placed in an incubator at 37 °C with 5% CO_2._ After 5 – 7 days, once the complete cytopathogenic effect (CPE) was observed for the positive control of 100 TCID_50_/50 μl, we recorded the level of cytopathogenic effect for each well. One sample for the neutralization assay in duplicate was observed simultaneously, and the assay should be repeated if the difference between the two wells was significant. For each serum sample, the reciprocal of the highest dilution of serum that could protect 50% of the cell well from CPE was defined as the SARS-CoV-2 NAb titer of that serum. The serum samples with titers above 1:4 were considered positive. We performed all procedures in a biosafety level-3 (BSL-3) laboratory.

### UPT-POCT and microneutralization assays for 812 serum samples from COVID-19 patients

To assess the correlation between the total RBD-specific antibody level and the NAb titers in COVID-19 patients, we collected 812 serum samples from 590 participants recruited from Guangdong CDC, including non-COVID-19 patients and 312 COVID-19 patients (213 recovered and 99 RP COVID-19 patients). Namely, some patients, especially the RP patients, had tested multiple times. Serum with a neutralizing titer greater than 1:4 was considered positive for neutralizing antibodies, and they were used for analyzing the correlation between T/C ratios and NAb titers. In a further analysis of the protective effects based on the 312 COVID-19 patients, only the lowest NAb titer of each patient was included for patients with two or more detection results. Then, we analyzed the correlations between T/C ratios and neutralizing titers, RP rates, ARP rates, and protective effects for these 312 cases.

### UPT-POCT and microneutralization assays for vaccinated individuals

Health individuals were vaccinated against COVID-19 and provided serum samples. These volunteers were immunized via two subcutaneous injections, separated by 28 days, of a COVID-19 vaccine (Sinovac, China). We collected serum samples 28 days after administering the second COVID-19 vaccine dose, and the samples with neutralizing titers 1:32, 1:48, 1:64, 1:96, 1:192 were further analyzed using both UPT-POCT and microneutralization assays.

## Supplementary Information

**Additional file 1.**

## Data Availability

The datasets supporting the conclusions of this article are included within the article and its additional file.

## References

[1] Wu Z, McGoogan JM (2020). Characteristics of and important lessons from the coronavirus disease 2019 (COVID-19) outbreak in China: summary of a report of 72314 cases from the Chinese Center for Disease Control and Prevention. JAMA..

[2] Zhao J, Yuan Q, Wang H, Liu W, Liao X, Su Y (2020). Antibody responses to SARS-CoV-2 in patients with novel coronavirus disease 2019. Clin Infect Dis.

[3] Chan J, Yuan S, Kok KH, To KK, Chu H, Yang J (2020). A familial cluster of pneumonia associated with the 2019 novel coronavirus indicating person-to-person transmission: a study of a family cluster. Lancet..

[4] Zhao Y, Zhang P, Wang J, Zhou L, Yang R (2020). A novel electro-driven immunochromatography assay based on upconversion nanoparticles for rapid pathogen detection. Biosens Bioelectron.

[5] Zhang P, Zhang Y, Zhao Y, Song Y, Niu C, Sui Z (2020). Calibration of an Upconverting phosphor-based quantitative Immunochromatographic assay for detecting *Yersinia pestis, Brucella* spp, and *Bacillus anthracis* Spores. Front Cell Infect Microbiol.

[6] Zhang P, Jiao J, Zhao Y, Fu MJ, Song YJ, Zhou DS (2020). Development and evaluation of an up-converting phosphor technology-based lateral flow assay for rapid and quantitative detection of Coxiella burnetii. BMC Microbiol.

[7] Liang Z, Wang X, Zhu W, Zhang P, Yang Y, Sun C (2017). Upconversion nanocrystals mediated lateral-flow Nanoplatform for in vitro detection. ACS Appl Mater Interfaces.

[8] Yu J, Tostanoski LH, Peter L, Mercado NB, McMahan K, Mahrokhian SH (2020). DNA vaccine protection against SARS-CoV-2 in rhesus macaques. Science..

[9] Chandrashekar A, Liu J, Martinot AJ, McMahan K, Mercado NB, Peter L (2020). SARS-CoV-2 infection protects against rechallenge in rhesus macaques. Science..

[10] Hansen J, Baum A, Pascal KE, Russo V, Giordano S, Wloga E (2020). Studies in humanized mice and convalescent humans yield a SARS-CoV-2 antibody cocktail. Science..

[11] Wu Y, Wang F, Shen C, Peng W, Li D, Zhao C (2020). A noncompeting pair of human neutralizing antibodies block COVID-19 virus binding to its receptor ACE2. Science..

[12] Shi R, Shan C, Duan X, Chen Z, Liu P, Song J (2020). A human neutralizing antibody targets the receptor-binding site of SARS-CoV-2. Nature..

[13] Li L, Zhang W, Hu Y, Tong X, Zheng S, Yang J (2020). Effect of convalescent plasma therapy on time to clinical improvement in patients with severe and life-threatening COVID-19: a randomized clinical trial. JAMA..

[14] Ravichandran S, Coyle EM, Klenow L, Tang J, Grubbs G, Liu S (2020). Antibody signature induced by SARS-CoV-2 spike protein immunogens in rabbits. Sci Transl Med.

[15] Hu F, Chen F, Ou Z, Fan Q, Tan X, Wang Y (2020). A compromised specific humoral immune response against the SARS-CoV-2 receptor-binding domain is related to viral persistence and periodic shedding in the gastrointestinal tract. Cell Mol Immunol.

[16] Kissler SM, Tedijanto C, Goldstein E, Grad YH, Lipsitch M (2020). Projecting the transmission dynamics of SARS-CoV-2 through the postpandemic period. Science..

[17] Wajnberg A, Amanat F, Firpo A, Altman DR, Bailey MJ, Mansour M (2020). Robust neutralizing antibodies to SARS-CoV-2 infection persist for months. Science..

[18] Yang C, Jiang M, Wang X, Tang X, Fang S, Li H (2020). Viral RNA level, serum antibody responses, and transmission risk in recovered COVID-19 patients with recurrent positive SARS-CoV-2 RNA test results: a population-based observational cohort study. Emerging Microbes & Infections.

[19] Yan Z, Zhou L, Zhao Y, Wang J, Huang L, Hu K (2006). Rapid quantitative detection of *Yersinia pestis* by lateral-flow immunoassay and up-converting phosphor technology-based biosensor. Sens Actuators B Chem.

[20] Zhang P, Gao Q, Wang T, Ke Y, Mo F, Jia R (2021). Development and evaluation of a serological test for diagnosis of COVID-19 with selected recombinant spike proteins. Eur J Clin Microbiol Infect Dis.

[21] Kitikoon P, Vincent AL (2014). Microneutralization assay for swine influenza virus in swine serum. Methods Mol Biol.

[22] Anderson LJ, Hierholzer JC, Bingham PG, Stone YO (1985). Microneutralization test for respiratory syncytial virus based on an enzyme immunoassay. J Clin Microbiol.

