# Supplementary material for: Development and multi-center clinical trials of an up-converting phosphor technology-based point-of-care (UPT-POCT) assay for rapid COVID-19 diagnosis and prediction of protective effects
Source: BMC Microbiol. 2022 Feb 3;22:42. doi: 10.1186/s12866-022-02450-z (PMC8811342; doi:10.1186/s12866-022-02450-z)
Supplement: Supplementary file 1 — Additional file 1. [file 12866_2022_2450_MOESM1_ESM.pdf]

Additional file 1

Development and clinical evaluation of an up-converting phosphor technology-based point-of-care (UPT-POCT) assay for diagnosis of COVID-19 and rapid prediction of protective effect

Pingping Zhang[1, 2#], Baisheng Li[3#], Yao Wang[4#], Wei Min[2#], Xiaohui Wang[5#], Yugui Zhou[1, 2#], Zhencui Li[3], Yong Zhao[1], Huan Zhang[3], Min Jiang[5], Huanying Zheng[3], Chao Yang[5], Wei Zhang[3], LeZuo[5], Qi Gao[2], Zhengrong Yang[5], Yanzhao Li[2], Tiejian Feng[5], Changqing Lin[2]*, Qinghua Hu[5]*, Tie Song[3]*, Ruifu Yang[1, 4]*

Author Affiliations:

[1]State Key Laboratory of Pathogen and Biosecurity, Beijing Institute of Microbiology and Epidemiology, Beijing 100071, P. R. China

[2] Beijing Key Laboratory of POCT for Bioemergency and Clinic (No. BZ0329), Beijing 100071, P. R. China

[3] Guangdong Provincial Center for Disease Control and Prevention, Guangzhou 511430, P. R. China

[4] Department of Clinical Laboratory, Peking Union Medical College Hospital, Peking Union Medical College, Chinese Academy of Medical Sciences, Beijing, 100032, People's Republic of China

[5]Shenzhen Center for Disease Control and Prevention, Shenzhen, P. R. China

#: These authors contributed equally to this work.

***Address for Correspondence**

Ruifu Yang, Beijing Institute of Microbiology and Epidemiology, No. 20, Dongdajie Street, Fengtai District, Beijing 100071, P. R. China. Email: ruifuyang@gmail.com

Tie Song, Guangdong Provincial Center for Disease Control and Prevention, Guangzhou 511430, P. R.

China. Email: sjkzx_songtie@gd.gov.cn

Qinghua Hu, Shenzhen Center for Disease Control and Prevention, Shenzhen, P. R. China. Email: huqinghua@szcdc.net

Changqing Lin, Beijing Key Laboratory of POCT for Bioemergency and Clinic (No. BZ0329), Beijing 100071, P. R. China. Email: changqing.lin@hotgen.com.cn

Supplementary Tables

Table S1 Detection results for the serum sample from one convalescent COVID-19 patient.

Sample size	Dilution fold	T/C ratio of UPT		OD value of ELISA	
		MEAN	SD	MEAN	SD
3	Negative control	0.175	0.015	1.00E-03	0.004
3	3200	0.211	0.006	0.009	0.007
3	1600	0.263	0.02146	0.033	0.00436
6	800	0.343	0.0097	0.084	0.01137
3	400	0.512	0.04277	0.164	0.007
3	200	0.761	0.05735	0.411	0.04291
3	100	1.45	0.09949	0.781	0.16901
3	50	3.09	0.34314	1.956	0.30453
3	25	4.291	0.07657	2.622	0.08271
3	12.5	8.154	0.264	2.794	0.01931
2	6.25	12.881	0.544	2.8835	0.05303

Table S2 Detection results for 39 serum samples taken from 27 COVID-19 patients during the acute phase of illness

Sample No.	Dilution Fold	T/C ratio of UPT		OD value of ELISA		Calculated T/C ratio*
		MEAN	SD	MEAN	SD	
**1**	100	1.417	0.089	0.685	0.136	1.274
**2**	25	0.769	0.092	0.297	0.048	0.673
**3**	100	0.738	0.091	0.577	0.062	1.107
**4**	400	1.245	0.01	0.661	0.061	1.237
**5**	100	1.253	0.109	0.8	0.071	1.452
**6**	100	0.672	0.028	0.89	0.105	1.592
**7**	100	2.279	0.259	2.167	0.202	3.57
**8**	400	2.582	0.017	0.419	0.042	0.862
**9**	100	0.83	0.14	0.743	0.061	1.364
**10**	100	0.902	0.128	0.585	0.098	1.119
**11**	100	0.376	0.03	0.178	0.007	0.489
**12**	400	0.978	0.027	0.747	0.047	1.37
**13**	100	2.546	0.098	2.132	0.051	3.515
**14**	400	1.483	0.082	1.452	0.088	2.462
**15**	400	1.04	0.046	0.433	0.006	0.884
**16**	100	1.473	0.047	0.73	0.078	1.344
**17**	100	3.51	0.179	1.286	0.328	2.205
**18**	100	0.73	0.048	0.34	0.018	0.74
**19**	100	2.859	0.102	1.13	0.059	1.963

**20**	100	1.147	0.112	1.109	0.052	1.931
**21**	100	1.455	0.071	1.417	0.122	2.408
**22**	100	3.487	0.112	0.714	0.064	1.319
**23**	100	2.062	0.157	0.682	0.07	1.269
**24**	100	1.847	0.065	1.176	0.25	2.035
**25**	100	1.024	0.087	0.59	0.077	1.127
**26**	100	3.374	0.264	2.046	0.211	3.382
**27**	100	1.71	0.124	1.401	0.206	2.383
**28**	100	0.956	0.092	0.49	0.017	0.972
**29**	100	2.341	0.33	1.617	0.104	2.718
**30**	100	0.279	0.021	0.079	0.002	0.335
**31**	100	0.648	0.033	0.667	0.02	1.246
**32**	100	3.376	0.259	2.119	0.105	3.495
**33**	100	1.895	0.212	1.785	0.044	2.978
**34**	400	1.221	0.003	1.052	0.129	1.843
**35**	100	0.363	0.027	0.266	0.018	0.625
**36**	100	1.732	0.112	0.893	0.033	1.596
**37**	400	1.449	0.073	0.883	0.013	1.581
**38**	100	0.702	0.074	1.53	0.086	2.583
**39**	100	1.066	0.06	1.798	0.036	2.998

[*] Calculated T/C ratio (y) = 1.549x + 0.213, where "x" is the OD value from an ELISA. This equation was obtained from the results of the convalescent serum samples.

Table S3 Correlation between the T/C ratios and NAb titers for 528 serum samples from 312 COVID-19 patients

Sample size	Neutralizing titer	MEAN of T/C	SD of T/C
16 (+1*)	1:4	0.859	0.669
46	1:8	1.166	0.846
54	1:16	1.901	1.073
140	1:32	2.735	1.860
75	1:64	4.062	2.450
103	1:128	5.019	2.941
40	1:256	5.917	3.335
33	1:512	7.230	2.936
14	1:1024	8.345	4.539
6	>1:1024	13.753	3.894
Total：528			

Note: One serum sample with neutralizing titer of 1:4 and T/C ratio of 38.54 was excluded for calculation of average of T/C, but included in Spearman rank correlation analysis.

Table S4 Correlation between the T/C ratios and RP rates

Neutralizing titer	1:4	1:8	1:16	1:32	1:64	1:128	1:256	1:512	1:1024
NRP cases	9	20	32	42	31	41	22	8	8
RP cases	6	12	12	35	10	16	5	3	0
Total	15	32	44	77	41	57	27	11	8
RP rate	40%	38%	27%	45%	24%	28%	19%	27%	0%
Mean of T/C ratio	0.738	1.246	1.960	3.287	**4.380**	5.546	6.204	6.339	9.689
SD of T/C ratio	0.509	0.914	1.143	2.102	**2.677**	3.033	3.710	1.414	5.730

SD: Standard deviation

Table S5 Correlation between the T/C ratios and accumulated RP (ARP) rates

Neutralizing titer	Accumulated NRP cases (a)	ARP cases (b)	Cases included (a+b)	ARP rate (b/a+b)	Cases in proportion ((a+b）/312）	MEAN of T/C ratio	SD of T/C ratio
≥1:4	213	99	312	32%	100%	0.738	0.509
≥1:8	204	93	297	31%	95%	1.246	0.914
≥1:16	184	81	265	31%	85%	1.960	1.143
≥1:32	152	69	221	31%	71%	3.287	2.102
≥1:64	110	34	144	24%	46%	4.380	2.677
≥1:128	79	24	103	23%	33%	5.546	3.033
≥1:256	38	8	46	17%	15%	6.204	3.710
≥1:512	16	3	19	16%	6%	6.339	1.414
≥1:1024	8	0	8	0%	3%	9.689	5.730

SD: Standard deviation

Table S6 Correlation between the T/C ratios and protection rates against RP

Neutralizing	Total – Accumulated	Protection rate	MEAN of	SD of T/C

titer	RP		T/C ratio	ratio
≥1:4	213	0.68	0.738	0.509
≥1:8	219	0.7	1.246	0.914
≥1:16	231	0.74	1.96	1.143
≥1:32	243	0.78	3.287	2.102
≥1:64	278	0.89	4.38	2.677
≥1:128	288	0.92	5.546	3.033
≥1:256	304	0.97	6.204	3.71
≥1:512	309	0.99	6.339	1.414
≥1:1024	312	1.00	9.689	5.73

SD: Standard deviation

Protection rate: (Total − Accumulated RP)/Total

Table S7 Detection results for the serum samples from COVID-19-vaccinated people

**Sample size**	**Neutralizing titer**	**UPT-POCT assay**	
		**MEAN of T/C ratio**	**SD**
2	1:32	1.327	0.047
13	1:48	2.275	1.438
5	1:64	2.354	0.608
8	1:96	4.345	1.525
7	1:192	8.177	3.492
Total: 35			
